# Supplementary material for: Simulating Hypoxia in a New England Estuary: WASP8 Advanced Eutrophication Module (Narragansett Bay, RI, USA)
Source: Water (Basel). Author manuscript; Available in PMC 2024 Mar 20. (PMC10077877; doi:10.3390/w15061204)
Supplement: Supplement1 [file NIHMS1885699-supplement-Supplement1.zip › water-2274888-supplementary.pdf]

Supplementary Materials

Simulating Hypoxia in a New England Estuary: WASP8 Advanced Eutrophication Module (Narragansett Bay, RI, USA)

Christopher D. Knightes

S1. Model Input

S1.1. Tributary Inflow

There are 8 tributaries with inflow to the Bay. Seven tributaries have USGS gauges (http://waterdata.usgs.gov/RI/nwis/current?type=flow). Because Warren River does not have a USGS gauge, the flow was estimated by adjusting the flow from a nearby river (Ten Mile River) with the ratio of their watershed drainage areas. Flows from riparian sub-watersheds are estimated by area and average flow per unit area from the gauged watershed area. Half of this flow is distributed equally in the Pawtuxet, Taunton, Hung, and Warren Rivers. The other half of the flow is distributed to central segments in the West Passage, East Passage and Sakonnet River. Based on salinity simulations and dye study observations, flow is added to the top and second layer of the model. Effluent flow for Bucklin Point and Field's Point WWTPs came from the Narragansett Bay Commission (NBC, https://www.narrabay.com/). Flow rates for Bristol, East Providence, East Greenwich, Jamestown, Newport, Quonset, Warren, Fall River, and Somerset WWTPs are from the US EPA's Integrated Compliance Information System (ICIS, https://www.epa.gov/enviro/pcs-icis-search).

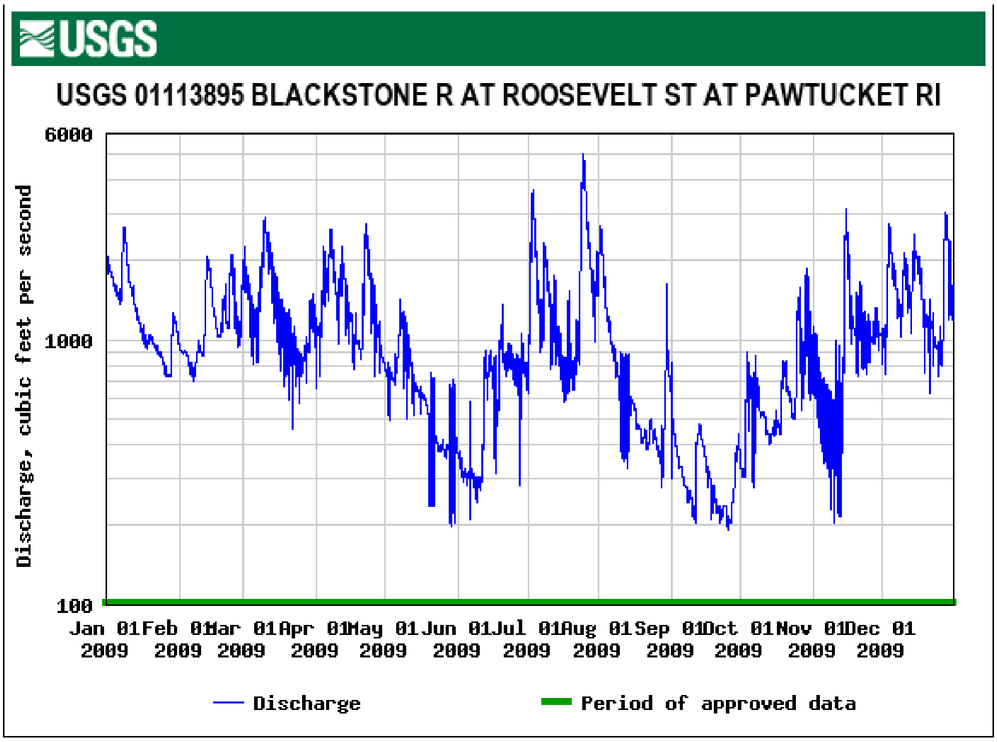

Figure S1. USGS tributary flow for Blackstone River, RI, US, which is the incoming tributary just above the Phillipsdale sample location (https://nwis.waterdata.usgs.gov/usa/nwis/uv/?cb_00060=on&cb_00065=on&format=gif_default&site_no=01113895&legacy=1&period=&begin_date=2009-01-01&end_date=2009-12-31, accessed 7 September 2018)

S1.2. Tributary Boundary Conditions

For all the tributary inflows, boundary concentrations were obtained from the NBC database (http://snapshot.narrabay.com/app/WaterQualityInitiatives/NutrientMonitoring), which include nitrate plus nitrite nitrogen (NO3+NO2 - N), ammonia nitrogen (NH4 - N), dissolved inorganic phosphorous (DIP), total dissolved nitrogen (TDN), total suspended solids (TSS), and water temperature. No data were available for particulate organic nitrogen (PON), dissolved organic phosphorus (DOP), or particulate organic phosphorous (POP), thus low concentrations were assumed. Dissolved Oxygen (DO) was set to saturation, calculated from temperature. Phytoplankton (chl *a*) were set to the average of New England freshwater streams [20]. There are 11 WWTPs that discharge into the Bay. For each of these, measured concentrations of nutrients, TSS, and ultimate carbonaceous biochemical oxygen demand (CBODU) were used as boundary conditions. Data for Bucklin Point and Field's Point WWTPs were provided by NBC. For the remaining nine, data were obtained from US EPA's ICIS. Nutrient concentrations at the seaward boundary were obtained from surveys conducted by the University of Rhode Island's Graduate School of Oceanography (URI GSO) and the Narragansett Bay Coastal Hypoxia Research Program (CHRP) [32].

Table S1. Ammonia (as N).

Date	Hour	Concentration [mg l^{-1}]
1/2/09	0:00:00	0.012
3/19/09	0:00:00	0
4/16/09	0:00:00	0.001
5/30/09	0:00:00	0
6/16/09	0:00:00	0.006
6/29/09	0:00:00	0
7/28/09	0:00:00	0.006
8/11/09	0:00:00	0.006
8/17/09	0:00:00	0.00322
9/15/09	0:00:00	0.00448
9/28/09	0:00:00	0.00616
10/18/09	0:00:00	0.00644
12/31/09	0:00:00	0.01736

Table S2. Nitrate + Nitrite (as N).

Date	Hour	Concentration [mg l^{-1}]
1/2/09	0:00:00	0.035
3/19/09	0:00:00	0.002
4/16/09	0:00:00	0.001
5/30/09	0:00:00	0.001
6/16/09	0:00:00	0.002
6/29/09	0:00:00	0.001
7/28/09	0:00:00	0.001
8/11/09	0:00:00	0.002
8/29/09	0:00:00	0.003276
9/9/09	0:00:00	0.007336
11/5/09	0:00:00	0.015008
12/31/09	0:00:00	0.02282

Table S3. Dissolved Organic Nitrogen (as N).

Date	Hour	Concentration [mg l^{-1}]
1/2/09	0:00:00	0.048
3/19/09	0:00:00	0.06
4/16/09	0:00:00	0.039
5/30/09	0:00:00	0.028
6/16/09	0:00:00	0.096
6/29/09	0:00:00	0.036
7/28/09	0:00:00	0.079
8/11/09	0:00:00	0.077
8/18/09	0:00:00	0.214249
9/16/09	0:00:00	0.133145
10/14/09	0:00:00	0.021374

11/16/09	0:00:00	0.067051
12/31/09	0:00:00	0.067051

Table S4. Dissolved Organic Nitrogen (as N).

Date	Hour	Concentration [mg l^{-1}]
1/2/09	0:00:00	0.028
3/19/09	0:00:00	0.016
4/16/09	0:00:00	0.012
5/30/09	0:00:00	0.013
6/16/09	0:00:00	0.016
6/29/09	0:00:00	0.014
7/28/09	0:00:00	0.016
8/11/09	0:00:00	0.006
8/17/09	0:00:00	0.0177
9/15/09	0:00:00	0.0159
9/28/09	0:00:00	0.0228
10/18/09	0:00:00	0.0315
12/11/09	0:00:00	0.0312
12/31/09	0:00:00	0.0312

Table S5. Dissolved Organic P (as P).

Date	Hour	Dissolved Organic P (as P) [mg l^{-1}]	CBOD (CBOD-1) [mg l^{-1}]	BOD (CBOD-2) [mg l^{-1}]
1/2/09	0:00:00	0.001	0.001	0.001
12/31/09	0:00:00	0.001	0.001	0.001

Table S6. Dissolved Oxygen.

Date	Hour	Concentration [mg l^{-1}]
1/2/09	0:00:00	10.0124
1/31/09	0:00:00	11.4011
2/1/09	0:00:00	11.4263
2/28/09	0:00:00	11.0375
3/1/09	0:00:00	11.0836
3/19/09	0:00:00	11.2648
3/31/09	0:00:00	10.5483
4/1/09	0:00:00	10.4598
4/16/09	0:00:00	10.6088
4/30/09	0:00:00	9.61646
5/1/09	0:00:00	9.35729
5/31/09	0:00:00	9.02927
6/1/09	0:00:00	8.43844
6/16/09	0:00:00	8.70125
6/30/09	0:00:00	7.8476

7/1/09	0:00:00	7.52573
7/31/09	0:00:00	6.39135
8/1/09	0:00:00	6.47823
8/31/09	0:00:00	6.42177
9/1/09	0:00:00	6.87344
9/17/09	0:00:00	7.26656
9/30/09	0:00:00	6.96469
10/1/09	0:00:00	7.06458
10/14/09	0:00:00	7.50948
10/31/09	0:00:00	8.32625
11/1/09	0:00:00	8.19135
11/30/09	0:00:00	9.69927
12/1/09	0:00:00	9.75698
12/29/09	0:00:00	9.05604
12/31/09	0:00:00	9.0576

Table S7. Detrital C (as C).

Date	Hour	Detrital C (as C) [mg l^{-1}]	Detrital N (as N) [mg l^{-1}]	Detrital P (as P) [mg l^{-1}]
1/2/09	0:00:00	0.001	0.001	0.001
12/31/09	0:00:00	0.001	0.001	0.001

Table S8. Dissolved Oxygen.

Date	Hour	Concentration [mg l^{-1}]
1/2/09	0:00:00	1.03542
1/31/09	0:00:00	1.04
2/1/09	0:00:00	1.66
2/28/09	0:00:00	1.65677
3/1/09	0:00:00	1.75104
3/19/09	0:00:00	2.05104
3/31/09	0:00:00	1.84687
4/1/09	0:00:00	1.89583
4/16/09	0:00:00	2.4875
4/30/09	0:00:00	2.91146
5/1/09	0:00:00	3.00521
5/31/09	0:00:00	2.31146
6/1/09	0:00:00	2.17917
6/16/09	0:00:00	3.675
6/30/09	0:00:00	3.25208
7/1/09	0:00:00	4.48021
7/31/09	0:00:00	5.2974
8/1/09	0:00:00	5.90938
8/31/09	0:00:00	3.79063

9/1/09	0:00:00	4.42396
9/17/09	0:00:00	5.05417
9/30/09	0:00:00	3.79479
10/1/09	0:00:00	3.85938
10/14/09	0:00:00	1.84063
10/31/09	0:00:00	1.06354
11/1/09	0:00:00	0.941667
11/30/09	0:00:00	1.675
12/1/09	0:00:00	1.63125
12/29/09	0:00:00	1.71667
12/31/09	0:00:00	1.4375

S1.3 Benthic Flux

Benthic fluxes of nutrients and oxygen were incorporated into the model as nitrate + nitrite, ammonia, and inorganic phosphate fluxes and sediment oxygen demand based on observations in the Providence River, Poppasquash Point, and Greenwich Bay [1].

S1.4 Atmospheric Deposition

Monthly atmospheric deposition data of nitrate were obtained from NTN-CT15 (Abington, CT, Latitude 41.8400, Longitude -72.0101, from National Atmospheric Program http://nadp.slh.wisc.edu/). Time series wind speed, solar radiation, and air temperature collected using EPA's HMS: Hydrologic Micro Services available at https://qed.eps.gov/hms. Wind Speed and Air Temperature were obtained from the Newport, RI Station 72507914787 (41.49 °N 71.31 °W). Wind Speed (m s^{-1}) and Air Temperature (°C) data are approximately twice per hour. Solar Radiation was obtained from North American Land Data Assimilation System (NLDAS), which provides total solar radiation at the water surface in Watts per square meter every hour (W m^{-2} h^{-1}) including ultraviolet, visible, and infrared wavelengths.

Table S9. Nitrogen Deposition.

Date	Atmospheric Deposition of Nitrate [mg m^{-2} d^{-1}]
12/30/08	0.202474
2/3/09	0.349746
3/3/09	0.461191
3/31/09	0.791029
4/28/09	1.258915
6/2/09	0.625204
6/30/09	1.768643
7/28/09	0.412529
9/1/09	0.300063
9/29/09	0.49571
11/3/09	0.31301

12/1/09	0.224243
12/29/09	0.225552
2/2/10	0.190149

S1.5 Meteorology

Wind speed, solar radiation, and air temperature collected using EPA's HMS: Hydrologic Micro Services available at https://qed.eps.gov/hms. Due to the large number of data points (every 30 minutes for wind and air temperature, and every hour for solar radiation), the time series are not provided here. They are readily accessible online.

Wind Speed and Air Temperature from Newport, RI Station 72507914787 (41.49° N 71.31° W). Wind Speed [m s^{-1}] and Air Temperature [°C] data are available approximately twice per hour.

Hourly Solar Radiation is from North American Land Data Assimilation System (NLDAS). NLDAS provides total solar radiation at the water surface in Watts per square meter every hour [W $m^{-2}h^{-1}$] including ultraviolet, visible, and infrared wavelengths.

Table S10. Light.

Constant	Value
Light Option	1 – Input diel light
Background Light Extinction	0.05 m^{-1}
Detritus & Solids Light Extinction Multiplier	0.2 m^{-1} (mg l^{-1})$^{-1}$
DOC Light Extinction Multiplier	0.2

S2. Observed Data

Monitoring data from the Fixed-Site Monitoring Network (FSMN) and the Narragansett Bay Commission (NBC) were used to compare to model simulation results at five locations: Phillipsdale, Bullock Reach, Conimicut Point, North Providence, Quonset Point (see Figure 2 for locations). The FMSN includes monitoring stations on buoys and shoreline installations throughout the bay, operated by the Rhode Island Department of Environmental Management's Water Resources Division, Narragansett Bay National Estuarine Research Reserve, and NBC. FMSN stations are mounted near the surface and the bottom and take samples every 15 minutes, between May or early June to late October. Parameters include depth, temperature, salinity, DO, and pH (http:/www.dem.ri.gov/bart). The NBC collects water quality profiles for the Upper Bay, including Phillipsdale, Bullock Reach, and Conimicut Point. Samples were monitored approximately every two weeks, including depth, temperature, and DO using a Seabird Electronics profiler (http://snapshot.narrabay.com/WaterQualityInitiatives/Profiles).

S3. Data Processing

Observed sonde data and WASP model output did not match in time and there were periods of no sonde data. MATLAB (MATLAB, 2021. v. 9.11.0 (R2021b)) code was written and used to process each set of data and average them per hour and remove data when there were gaps to provide a direct comparison of simulated to observed data.

References

[1] Fulweiler, R.W.; Nixon, S.W.; Buckley, B.A. Spatial and temporal variability of benthic oxygen demand and nutrient regeneration in an anthropogenically impacted New England estuary. *Estuaries Coasts* **2010**, *33*, 1377–1390.
